# Association between nutritional quality and the degree of naturalness in animal-based and plant-based food products

**DOI:** 10.1017/jns.2025.10056

**Published:** 2025-11-26

**Authors:** Dylan Guillemette, Marie-Ève Labonté, Sonia Pomerleau, Julie Perron, Alicia Corriveau, Mylene Turcotte, Véronique Provencher

**Affiliations:** 1 Centre Nutrition, santé et société (NUTRISS), Institute of Nutrition and Functional foods (INAF), Université Laval, Québec, QC, Canada; 2 School of Nutrition, Faculty of Agriculture and Food Science, Université Laval, Québec, QC, Canada

**Keywords:** Food naturalness, Food product package, Food supply, Nutritional quality, Plant-based foods, DSCF, Dwass, Steel, Critchlow-Fligner, FNI, Food Naturalness Index, FOP, Front-of-pack, FVNL, Fruit, vegetable, nut and legume, NP, Nutrient profiling

## Abstract

Consumers tend to perceive certain foods as more natural and in turn as more nutritious. Thus, this study aimed to evaluate the nutritional quality, the degree of naturalness, and their association with animal-based and plant-based food products. A total of 1275 food products were collected by the Food Quality Observatory in Québec (Canada) between 2018 and 2022. These products were divided into five categories: sliced processed meats (*n* = 477), yogurts and dairy desserts (*n* = 344), sausages (*n* = 266), processed cheese products (*n* = 96) and plant-based alternatives (*n* = 92) within these four categories. The overall nutritional quality was evaluated using the Nutri-Score and the front-of-package (FOP) nutrition symbol recently implemented in Canada, while the degree of naturalness was measured using the Food Naturalness Index (FNI). Yogurts and dairy desserts as well as plant-based alternatives had lower Nutri-Score and thus, higher nutritional quality compared to other food categories. The FOP symbol for foods high in saturated fat or sodium was more common in sliced processed meats and sausages. FNI scores were higher in processed cheese products than in other categories, indicating a greater degree of naturalness. Correlations between nutritional quality and food naturalness varied depending on the food category and the nutrient profiling model, with Spearman coefficients being positive or negative and ranging from weak to moderate. This study supports the idea that food naturalness and nutritional quality offer complementary information depending on the food category. Further research in other food categories would help to better understand the associations between the two concepts.

## Introduction

Throughout the 20^th^ century, food processing rapidly grew in importance. Whether it is for preservation purposes or the creation of new foods from existing primary resources, food processing remains to this day a key component in ensuring the sustainability of the food supply around the world.^(1,2)^

In recent years, consumers have expressed more and more interest in healthier foods as well as ingredients and processing labelled as ‘natural’ by manufacturers.^(3–5)^ Furthermore, the increasing development of foods made from plant-based proteins has contributed to the rise in popularity of plant-based diets.^(6,7)^ It has been demonstrated that diets incorporating a large number of minimally processed plant-based foods (e.g., fruits, vegetables, whole grains) is beneficial for human health.^(8)^ The emphasis on plant-based foods is further highlighted in the 2019 Canada’s Food Guide, the latter which also suggests limiting the consumption of all highly processed foods and drinks (e.g., chocolate and candies, potato chips, bakery products, sugary drinks).^(9)^ Indeed, nutrients of public health concern, such as added sugar, sodium and saturated fat, are often found in significant amounts in numerous processed foods.^(10,11)^ Overconsumption of these nutrients has been associated with higher risks of developing noncommunicable diseases, such as heart disease or cancer.^(12,13)^

However, plant-based substitutes often require long lists of ingredients to adequately replicate the sensory properties of their animal-based counterparts.^(14)^ Processed or highly processed foods such as plant-based alternatives can vary considerably in terms of nutritional composition,^(15–17)^ hence the interest in assessing their nutritional quality, especially in comparison to their animal-based counterparts. The overall nutritional quality of foods can be evaluated with a nutrient profiling (NP) model, which is used to identify and rank foods rich in beneficial nutrients (e.g., proteins, fibre, vitamins) and/or in nutrients that should be limited (e.g., sugar, sodium, saturated fat).^(18,19)^ Food processing may impact the nutritional quality of food products in different ways,^(20)^ but other aspects such as choices of ingredients or farming practices can also influence nutritional quality. All these aspects can also make foods appear less processed and more natural, contributing to their naturalness or lack thereof in the eyes of consumers. Thus, it appears necessary to study the association between nutritional quality and food naturalness.

Up to now, food naturalness remains a concept devoid of a clear and regulated definition in most countries, including Canada, which makes it more difficult to assess.^(5,21,22)^ In an attempt to evaluate the degree of naturalness more thoroughly in food products, the Food Naturalness Index (FNI) was developed in Europe.^(23)^ The FNI includes four component criteria (farming practice, number of additives, number of unnecessary/unexpected ingredients and number of processed ingredients) based on various attributes identified through previous research on the perception of consumers regarding food naturalness.^(5)^ Unlike classification systems such as NOVA,^(24)^ which rank food into various groups to characterize the degree of processing (e.g., minimally processed or ultra-processed), the FNI allows to evaluate the degree of naturalness of food products with the aforementioned criteria and the list of ingredients exclusively. The association between food naturalness and nutritional quality can thus be appropriately evaluated using both the FNI and a NP model, while only using the information available on the product label.

In that context, the present study aimed to evaluate the nutritional quality, the degree of naturalness and their association in food categories that include both plant-based and animal-based food products (i.e., processed dairy or meats). It is hypothesized that plant-based alternatives and processed dairy food products have higher nutritional quality compared to processed meats,^(25,26)^ due to their generally lower contents of saturated fat and sodium. In terms of naturalness, it is difficult to predict which food category would have the highest FNI score, and thus, would have the highest naturalness score. Due to the important variety of products found within each food category, especially in plant-based alternatives of the other categories, it is expected that standard deviations in FNI scores will be high among food categories. Finally, it is expected that nutritional quality will be independent of the degree of naturalness, as noted in previous research showing the lack of a strong correlation between the two concepts.^(27,28)^

## Methods

### Food composition data

This study used databases from the Food Quality Observatory located at INAF, *Université Laval*, Québec.^(29)^ Food products from the following categories were previously collected in supermarkets and grocery stores in Québec: sliced processed meats (*n* = 477, 2022),^(30)^ yogurts and dairy desserts (*n* = 344, 2018–2019),^(31)^ sausages (*n* = 266, 2019)^(32)^ and processed cheese products (*n* = 96, 2020).^(33)^ Of note, the yogurts and dairy desserts category also comprised products from the city of Montréal due to a collaboration with *Protégez-Vous,* a Québec-based non-profit organization specializing in consumer information and product testing. Since these food categories initially contained both animal-based and plant-based products, a fifth category was created in the context of the current study to bring together all plant-based alternatives that had been collected by the Food Quality Observatory within each of the four previous categories. More specifically, there were 11 sliced processed meat substitutes, 36 dessert substitutes, 23 plant-based sausages and 22 processed cheese substitutes (*n* = 92, 2018–2022). Overall, a total of 1275 food products were collected and analyzed for the purpose of this study. Photos of all sides of the food packages were taken to help compile various types of information, such as the name of both the product and the brand, the Nutrition Facts table and the ingredients list.

### Nutritional quality of food products

The overall nutritional quality of food products was evaluated using both the Nutri-Score NP model^(34)^ and the front-of-package (FOP) nutrition label regulations in Canada.^(35)^ The Nutri-Score was chosen for comparison purposes since it is the only NP model that has been used alongside the FNI so far in the scientific literature when evaluating the association between nutritional quality and food naturalness.^(23,27,28)^ Furthermore, the Nutri-Score generates more precise and continuous numerical scores as opposed to solely classifications, thus also enabling a larger number of analyses to be conducted. Determination of Nutri-Score values were conducted using double-coding in the 2017 Nutri-Score calculator made by *Santé Publique* of France.^(34)^ The Nutri-Score considers both nutrients to limit (e.g., saturated fats, sugar, sodium) and to encourage (e.g., proteins, fibres). Because points for nutrients to encourage are subtracted from points for nutrients to limit, Nutri-Score values range between -15 and 40, for which a lower score reflects a higher nutritional quality.^(36)^ Nutri-Score results were double-coded and validated by two of the authors (D.G. and M.T.). The application of a specific methodology developed by Vergeer *et al*. (2020) was required to consider the Canadian context^(37)^ when determining each product’s proportion of fruit, vegetable, nut and legume (FVNL), which is necessary for the calculation of the Nutri-Score. Indeed, the FVNL content is not available quantitatively in Canada on the ingredients list displayed on food packages. Therefore, estimation of the FVNL content must be performed using a points system. More specifically, depending on the identity of the first three ingredients found in the ingredients list of the food product, a certain number of points is assigned, which is then converted to an estimated proportion of FVNL in the Nutri-Score calculator.^(37)^

The Health Canada FOP nutrition symbol was also used in the analyses since it is currently the only government-based NP model in Canada that will soon be required on food packaging. The presence of the Canadian FOP nutrition symbol for nutrients of public health concern (sugar, saturated fat and/or sodium) was determined for each product individually using Health Canada’s labelling guide for industry.^(35)^ The FOP symbol was not yet available on the food packaging of the products collected by the Food Quality Observatory at the time of the study, because the industry must comply with the regulations as of January 1^st^, 2026.^(35)^ Health Canada developed the FOP symbol to indicate when food products are high in any of the three nutrients of public health concern based on specific thresholds. Thresholds for the FOP nutrition symbol vary depending on the reference amount or the serving size, whichever of the two is the highest. For the prepackaged food products analyzed in this study, thresholds for the three nutrients were either ≥10% (if reference/serving amount was ≤30 g or 30 ml) or ≥15% (if reference/serving amount was >30 g or 30 ml) of the daily value. Some exemptions may be found in specific cases for certain food products. For example, the FOP symbol for saturated fat can be omitted in certain cheese products if they also contain a sufficient amount of calcium.^(35)^ Results for the FOP symbol were double-coded and validated by D.G. and the team’s statistician.

### Adaptation of the FNI to the Canadian context and calculation of FNI scores

Preliminary tests revealed a few issues when trying to determine FNI scores on food products in Québec. Indeed, Canadian food regulations present some key differences compared to European food laws that can cause problems when calculating FNI scores. Therefore, adaptation of the FNI was necessary to ensure proper evaluation of the degree of naturalness of foods sold in Québec. The adaptations performed were discussed with researchers and experts from both food science and nutrition (see Acknowledgement section) during a discussion that aimed to approve changes to the original FNI tool for each of the four component criteria. In the end, the following adjustments were made:

1. **Farming practices**: Unlike the other criteria which are all evaluated on a scale of 1 to 5, the farming practices score instead ranges from 2 to 5 points. Originally, foods with a score of 3 or 5 points had to have a mention that they were pesticide-controlled,^(23)^ and this characteristic was limited to baby food grade products. However, no pesticide-controlled mentions exist in Canada, meaning that scores of 3 or 5 points are unobtainable, and no other information was identified as a relevant alternative. While this issue does not directly impact this study since no baby food grade products were analyzed, only scores of 2 (regular food) or 4 points (organic food) can be attributed thus far to foods in Canada.
2. **Additives**: Most food additives found in Europe are also present in the Lists of Permitted Food Additives in Canada.^(38)^ Nevertheless, the following ingredients are not classified as additives in Canada compared to Europe: modified starches, sodium caseinate, monosodium glutamate, konjac gum, disodium guanylate, and calcium inosinate. Following the discussion between researchers and experts from food science and nutrition, modified starches and konjac gum were finally considered as additives because of their functionality. Flavour enhancers (monosodium glutamate, disodium guanylate and calcium inosinate) and sodium caseinate were treated as both unnecessary and processed ingredients (see Tables 1 and 2 below).
  **Table 1.** List of unexpected/unnecessary ingredients adapted to the Canadian context

Unexpected/unnecessary ingredients	Examples	Exceptions
Thickeners	Native starches, maltodextrins, cellulose	**Fibre**, other thickeners considered as additives
Non-commercial oils and fats	Palm oil, safflower oil, hydrogenated fats and oils, oils and fats where the origin is not mentioned (e.g., vegetable oil)	–
Artificial flavours	Any flavour without a ‘natural’ mention must be considered as artificial	Natural flavours
Glucose/fructose syrups	Corn syrup, rice syrup, glucose/fructose syrup, isoglucose	–
Added sugar	Added sugar in cereals, meats, chips, etc.	Honey, added sugar in jams or desserts
Added salt	Added salt in desserts	Added salt in meat and cheese products
Dairy ingredients (only in non-dairy products)	**Milk, whey, milk ingredients, etc.**	–
Plant-based ingredients (only in animal-based products)	**Cereals (wheat, rice, etc.), plant-based proteins (e.g., gluten), fibres, etc.**	**Vegetable oils**
Baking ingredients (do not consider in desserts or baking products)	**Baking powder, flours, cocoa powder, etc.**	**Flours in plant-based alternatives to meat products**
Ingredients not considered as additives in Canada	**Monosodium glutamate, disodium guanylate, calcium inosinate and sodium caseinate**	**Konjac gum and modified starches.** **Do not consider sodium caseinate in dairy products or desserts**

*Note:* Ingredients in bold were not specifically indicated in the original list.
  **Table 2.** List of processed ingredients adapted to the Canadian context

Processed ingredients	Examples	Exceptions
Isolated ingredients	Fibre, native starches, milk ingredients, etc.	–
Refined oils and fats	Plant oils (olive, coconut, canola, etc.), animal fat (butter, lard), margarine	Cream, less refined oils (virgin olive oil, cold-pressed canola oil)
Flavours	Artificial or natural flavours	–
Refined sugar	Sugar and syrups (corn, maple, fructose, etc.)	Honey
Refined cereals, flours, and pasta	Cereal flour (wheat, rice, corn)	Claim of “wholegrain” in any flour, cereal, and pasta. Oat and barley because they are usually whole grain.
Hydrolyzed and extruded cereals	Hydrolyzed infant cereals, cereals snacks (puff corn, flakes)	Pasta is excluded
Processed powdered ingredients	**Nutritional yeast, baking powder, cocoa powder, flavour enhancers (monosodium glutamate, disodium guanylate, etc.)**	**Any other dehydrated, powdered or concentrated ingredient conceived with a simple water removal process**
Complex ingredients	**Cheese, processed meat (bacon, ham, etc.), sauces, etc.**	**Alcohol drinks (beer, wine and liquors)**

*Note:* Ingredients in bold were not specifically indicated in the original list.
3. **Unexpected/unnecessary ingredients**: Table 1 compiles unexpected or unnecessary ingredients that were proposed to complete the list developed for the original FNI.^(23)^ Ingredients in bold were not specifically indicated in the original list (e.g., dairy ingredients, baking ingredients, etc.). They were added in this study to add more precision to this criterion since originally, these ingredients were all simply noted as ‘Ingredients that are not expected to be in the product or recipe’ in the table.^(23)^
4. **Processed ingredients**: Similarly, Table 2 presents processed ingredients proposed for this study. A major difference between this adapted list and the original one^(23)^ is the replacement of ‘Dehydrated/concentrates and powdered ingredients’ by ‘Processed powdered ingredients’. In Canada, processes like dehydration, concentration (without chemical change) and drying are classified as ‘Minimum processes’ that affect ‘the natural character of foods with a minimum of physical, chemical or biological changes’.^(39)^ Consequently, only powdered ingredients that require multiple processing steps were considered as processed ingredients. Once again, ingredients in bold were not specifically indicated in the original list.

Using this adapted version of the FNI that properly considers food regulations in Canada, evaluation of the degree of naturalness was determined for all foods included in the five food categories under study using the ingredients list, which is mandatory on each of the prepackaged food products. Once the scores for all four criteria were established, the final FNI score was determined by calculating the mean score from the four criteria. All FNI scores were double-coded and validated by D.G. and two research assistants (see Acknowledgement section), with S.P. acting as a third-party in cases of disagreement.

### Statistical analyses

Kruskal-Wallis tests were performed to illustrate differences in Nutri-Score and FNI scores between food categories, while the Dwass, Steel, Critchlow-Fligner (DSCF) procedure was used to verify if those differences were statistically significant. Box plots were used to illustrate the different results. Lines extend from each box to show the range of Nutri-Score or FNI scores within a food category. Dots placed outside the boxes indicate outliers, while the horizontal line and diamond-shaped dot inside the boxes indicate median and mean scores respectively. Finally, the different letters shown above the boxes indicate a significant difference at p < 0.05 when applying the Dwass, Steel, Critchlow-Fligner procedure.

In addition, Spearman correlations were conducted to evaluate the association between nutritional quality (both in terms of Nutri-Score and the number of nutrients requiring the FOP symbol) and the degree of naturalness (FNI) within each food category. A *p*-value < 0.05 was considered significant. Statistical tests were performed using SAS Studio version 3.81.

## Results

**Fig. 1.**
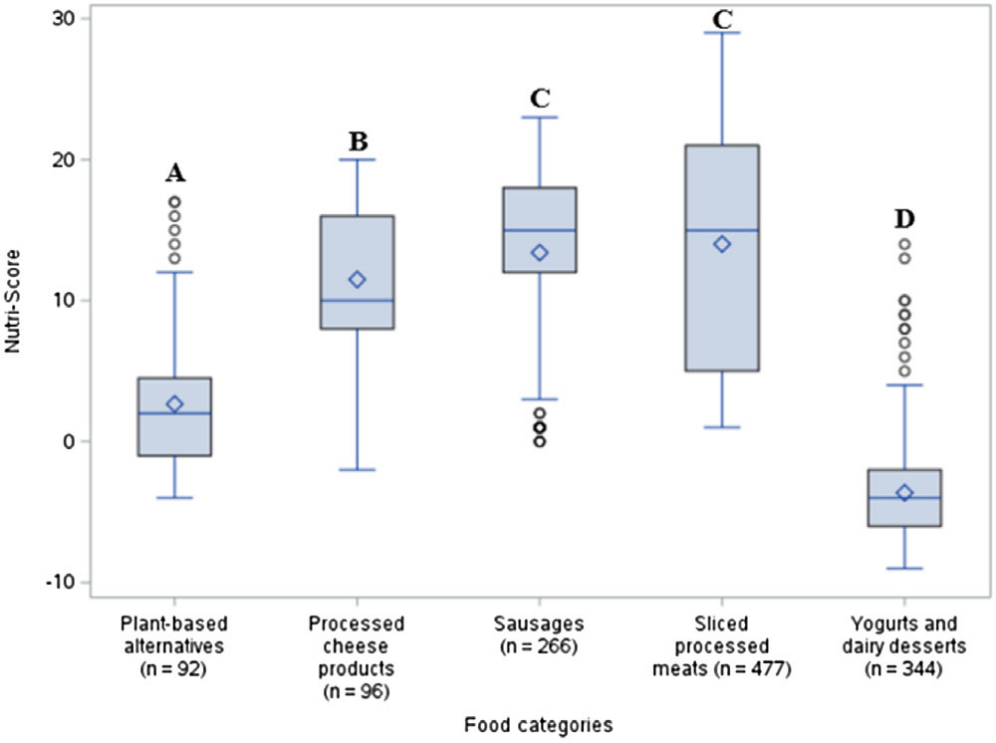
Comparison of the Nutri-Score values between the five food categories using the Kruskal-Wallis test.

Figure 1 presents the comparison of each food category’s overall nutritional quality, as assessed by the Nutri-Score NP model. Yogurts and dairy desserts had the lowest Nutri-Score mean value (−4 ± 3), reflecting a higher overall nutritional quality, followed by plant-based alternatives (3 ± 5) and then, processed cheese products (12 ± 5). Nutri-Score values were significantly different between categories (*p* < 0.0001), except between sliced processed meats and sausages (14 ± 8 and 13 ± 6, respectively, *p* = 0.31). These categories also had the highest Nutri-Score, meaning that they had a lower overall nutritional quality.

Regarding the FOP symbol, Table 3 shows that three of the five categories have 75% or more of their products carrying the symbol for at least one nutrient. More specifically, sausages and processed cheese products were the categories that had the most products with a FOP symbol for saturated fat, while the sodium FOP symbol was more often found in sliced processed meats and sausages. The symbol for sugar was mostly found in yogurts and dairy desserts. The FOP symbol for sodium or saturated fat was also present to a lesser extent in yogurts and dairy desserts as well as in plant-based alternative products.

**Table 3.** Presence of the health Canada’s front-of-pack (FOP) symbol for the three nutrients of concern among the five studied food categories

Food categories	Number of products	Saturated fat FOP symbol (number of products (%))	Sodium FOP symbol (number of products (%))	Sugar FOP symbol (number of products (%))	Number of products (%) with the FOP symbol for at least one nutrient
Plant-based alternatives	92	35 (38)	33 (36)	10 (11)	69 (75)
Processed cheese products	96	47 (49)	3 (3)	0 (0)	47 (49)
Sausages	266	198 (74)	233 (88)	0 (0)	239 (90)
Sliced processed meats	477	224 (47)	461 (97)	0 (0)	462 (97)
Yogurts and dairy desserts	344	15 (4)	1 (0.3)	105 (31)	106 (31)
**Total**	**1275**	**519 (41)**	**731 (57)**	**115 (9)**	**923 (72)**

**Fig. 2.**
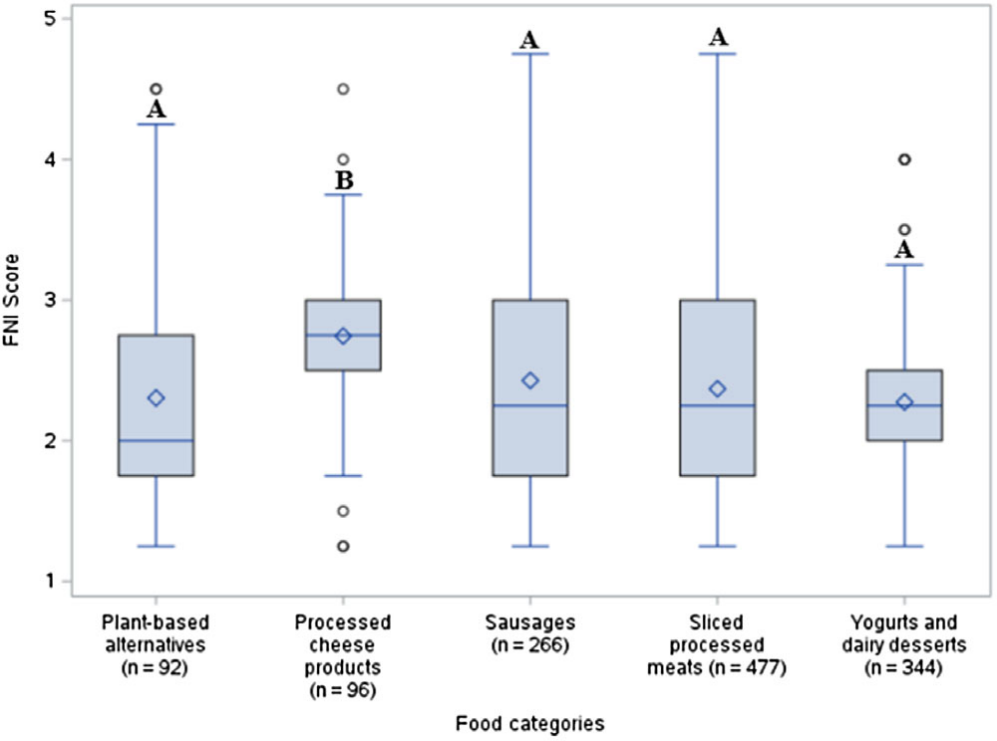
Comparison of FNI scores between the five food categories using the Kruskal-Wallis test.

Figure 2 illustrates the comparison of the degree of naturalness between food categories, as assessed by the FNI. FNI scores were only higher in processed cheese products (2.74 ± 0.61) compared to sliced processed meats (2.37 ± 0.87; *p* < 0.0001), yogurts and dairy desserts (2.28 ± 0.49; *p* < 0.0001), sausages (2.43 ± 0.98; *p* = 0.0003) and plant-based alternatives (2.30 ± 0.88; *p* < 0.0001). This means that processed cheese products had a higher degree of naturalness compared to the other food categories analyzed. No differences were observed in FNI scores between the other food categories.

Significant correlations between the overall nutritional quality, as evaluated by the Nutri-Score, and the degree of naturalness, as determined with the FNI, were observed in the following four categories: processed cheese products, sausages, sliced processed meats and yogurts and dairy desserts (Table 4). The correlations were negative for processed cheese products, sausages, as well as yogurts and dairy desserts, which means that a higher degree of naturalness was associated with a lower Nutri-Score (i.e., a better nutritional quality). Inversely, the sliced processed meats category was the only one with a positive association between both variables, indicating that a higher degree of naturalness is associated with a higher Nutri-Score (i.e., a lesser nutritional quality).

**Table 4.** Spearman correlations between overall nutritional quality and the degree of naturalness in the studied food categories

Food categories	Correlation between Nutri-Score and FNI*		Correlation between number of nutrients requiring FOP symbol and FNI*	
	rs	*p*-value	rs	*p*-value
Plant-based alternatives (*n* = 92)	−0.16	0.14	−0.42	<0.0001
Processed cheese products (*n* = 96)	−0.48	<0.0001	0.31	0.002
Sausages (*n* = 266)	−0.24	<0.0001	−0.02	0.76
Sliced processed meats (*n* = 477)	0.10	0.03	0.00	0.98
Yogurts and dairy desserts (*n* = 344)	−0.17	0.002	−0.32	<0.0001

*Note:* * Food Naturalness Index.

Significant correlations between naturalness and overall nutritional quality, as assessed by the number of nutrients requiring the FOP symbol, were observed in the three following categories: plant-based alternatives, processed cheese products and yogurts and dairy desserts (Table 4). The correlations were negative for plant-based alternatives and yogurts and dairy desserts, indicating that a lower number of nutrients requiring the FOP symbol (i.e. higher nutritional quality) was related to a higher degree of naturalness in these two food categories. On the other hand, the processed cheese products category showed a positive association between both variables, which means that a higher number of nutrients requiring the FOP symbol (i.e. lower nutritional quality) is related to a higher degree of naturalness.

## Discussion

The current study evaluated the nutritional quality, the degree of naturalness and their association in both animal-based and plant-based food products, to verify whether processed foods with a higher naturalness score would also have a higher overall nutritional quality. Nutritional quality, as evaluated by the Nutri-Score and Canadian FOP regulations, and the degree of naturalness, as measured by the FNI, varied between the studied food categories, namely sliced processed meats, yogurts and dairy desserts, sausages, processed cheese products and plant-based alternatives.

### Nutritional quality

Regarding nutritional quality, significant differences were observed between the five food categories. As hypothesized, yogurts and dairy desserts in addition to plant-based alternatives presented the highest nutritional quality as determined by the Nutri-Score. This can be explained by their lower content of saturated fats and sodium compared to processed cheese products, sliced processed meats and sausages. This is partially reflected by the fewer number of products requiring the FOP symbol in yogurts and dairy desserts and in plant-based alternatives, but only when comparing with sliced processed meats and sausages. Indeed, cheese products do not have to carry the FOP symbol for sodium and saturated fat if they contain a sufficient amount of calcium (>5% of the daily value)^(40)^ and if they do not contain certain ingredients with added saturated fats.^(35)^ This explains why processed cheese products have the second lowest amount of products that would require the symbol for at least one nutrient. Although yogurts and dairy desserts had the most products with a FOP symbol for sugar, it is only around 31% of those products that would actually carry this symbol, which further explains why Nutri-Score results tend to be lower in that food category. These findings are similar to those found in studies by Ovrebo *et al.* ^(41)^ and Ter Borg *et al.*,^(25)^ where Nutri-Score values were lower in some dairy products like yogurts compared to meat products. On the other hand, cheese products in both studies tended to have higher Nutri-Score due to their high amount of saturated fats and sodium. In the current study, only cheese products receiving further processing were evaluated (e.g., cream cheese, cheese slices, spreadable cheese), yet results remain similar to the cited studies. Indeed, processed cheese products were often rich in saturated fat and/or in sodium, which could explain their higher Nutri-Score scores. As such, the processed cheese product category had lower nutritional quality compared to plant-based alternatives, yogurts, and dairy desserts. As for sliced processed meats and sausages, they tend to have even higher amounts of both saturated fat and sodium, which lead to higher Nutri-Score results indicating poorer nutritional quality. The high quantity of processed meat products with the FOP symbol for saturated fat can be explained by the abundance of products that use red meat (e.g., beef, pork) in their formulations. Compared to poultry, red meat contains higher amounts of saturated fat.^(42)^ In order to substitute saturated fats found in common processed red meats and avoid the FOP symbol for this nutrient, food manufacturers may add plant-based ingredients such as vegetable oils, starches and fibre.^(43,44)^ Finally, the sodium content remains high across most processed meats due to its role in taste, texture and preservation.^(45,46)^ The use of potassium salts or ingredients with antimicrobial attributes like garlic or celery powder^(45,47)^ remain possible strategies to reduce, at least partially, the sodium content in these types of food, as seen by the few processed meats with no FOP symbol for sodium. Finally, the high proportion of products with at least one nutrient requiring the FOP symbol highlights the necessity to reformulate these food categories. Most notably, reducing the content in sodium and saturated fat would improve the quality of the food supply for those categories.

### Degree of naturalness

Food categories from the current study showed similar degrees of naturalness, except for a higher mean FNI score in processed cheese products. This can be attributed to the abundance of cream cheese products in this category, which tend to have shorter ingredients lists with fewer unnecessary and/or processed ingredients, thus leading to a higher FNI score. As hypothesized, variations of the FNI scores were high, especially in sliced processed meats, sausages, and plant-based alternatives. This could be explained by the large variety of food products in those categories, with some products like prosciutto or plant-based cream cheeses having a short list of ingredients with few additives or processed ingredients, while others like bologna, hot dog sausages and most plant-based meat substitutes having low FNI scores because of their long lists of ingredients. In plant-based substitutes, the need for a larger list of ingredients that often contain multiple additives can be partly attributed to the functionality of plant-based proteins. Unappealing colours, bitter taste and structural differences that hinder texture mimicking of animal-based products are all challenges that can be encountered when working with plant-based proteins.^(48–50)^ Consequently, the use of additives such as gums, emulsifiers or colouring agents is often employed to compensate for the shortcomings of plant-based proteins. Despite this, FNI scores in plant-based alternatives often remain similar when compared to the other food categories with animal-based products. Conversely, as the name suggests, most of the yogurts and dairy desserts category is comprised of yogurts, which have similar ingredients across the different possible types (e.g., ready-to-drink, skyr, Greek). This relative homogeneity among yogurt products could explain why the variability for FNI scores is lower in this category compared to processed meats and plant-based alternatives.

### Association between nutritional quality and food naturalness

In contrast to our hypothesis, findings from this study imply that food naturalness and nutritional quality may be related concepts, at least for some of the food categories that were analyzed. However, the association between nutritional quality and food naturalness appears to vary depending on the food category. In most categories, correlation results were negative, indicating that foods with higher Nutri-Score and thus lower nutritional quality would be less natural on the FNI scale. On the other hand, the opposite is observed in sliced processed meats, where foods with higher FNI scores also have higher Nutri-Score scores and thus poorer nutritional quality, oftentimes due to their high saturated fat and sodium content. The association between nutritional quality and food naturalness appears to also change depending on the NP model used for evaluating nutritional quality. Indeed, while a moderate negative association between food naturalness and nutritional quality as determined by the Nutri-Score was found in processed cheese products, the association became positive and weaker when nutritional quality was assessed instead by the number of nutrients requiring the FOP symbol. This can be attributed to the fact that NP models may measure different aspects of nutritional quality. In this case, the Nutri-Score accounts for both nutrients to encourage and nutrients to limit,^(34)^ while the FOP nutrition symbol proposed by Health Canada only takes nutrients to limit into consideration.^(35)^ Since the FOP symbol in processed cheese products is mostly present for saturated fat, this positive association implies that products with a high amount of saturated fat tend to have a higher degree of naturalness. A possible explanation could be that lighter versions of processed cheese products may employ additional ingredients, mostly additives such as gums, to compensate for the loss of creaminess, richness and mouthfeel due to the presence of fat, thus lowering their FNI scores.

These findings implicating that food naturalness may be a concept related to nutritional quality in select food categories are not in line with previous research. Abreu and Liz Martins (2023) compared the classification by the NOVA system of various food products on the Portuguese market with their nutritional quality using the Nutri-Score.^(16)^ Significant but often weak correlations were observed between the two variables, suggesting that nutritional quality and food processing are not associated, nor are they predictors of one or the other, although it is important to remember that food processing and food naturalness are not the same. Studies measuring the degree of naturalness with the FNI have also concluded that the FNI and NP models like the Nutri-Score measure different aspects of food products, as indicated by the weak correlation observed between both concepts.^(27,28)^ The association between food naturalness and nutritional quality can be indeed complex. For example, adding plant-based ingredients such as vegetable oils or fibre can help improve nutritional quality in meat products, despite the fact that these ingredients are considered unexpected and/or processed by the FNI framework. On the other hand, having a smaller ingredients list with few ingredients may improve the FNI score of a food product (as seen in some organic sliced processed meats, for example), but if those ingredients are inherently rich in saturated fat, sodium and/or sugars, then the nutritional quality will be lesser regardless. Further research is warranted in the future with other food categories to better understand the association between food naturalness and nutritional quality. In the meantime, nutritional quality does not seem to be totally independent of food naturalness, at least in the current analyzed food categories.

### Strengths and limitations

The main strength of this study is the analysis of food naturalness in food categories such as processed meats, dairy products and plant-based alternatives, which have never been analyzed in the scientific literature to date using the FNI tool. The advantage of using the FNI over other classification methods based on food processing such as NOVA is the possibility of better observing differences in naturalness, not only between food categories but within a category as well. The FNI also has four relatively well-defined criteria ensuring proper food naturalness evaluation. Furthermore, the data used in this study represented around 80% of all sliced processed meats, yogurts and dairy desserts, sausages and processed cheese products (including any plant-based alternatives) available in Québec between 2018 and 2022. Nonetheless, some limitations do exist in this research project. As mentioned earlier, the information on the FVNL content in food, which is necessary for determining Nutri-Score scores, is not available on food packaging in Canada. Therefore, the FVNL content remains an estimation of its real proportion, even if done in a double-coding format. The most recent version of the Nutri-Score (updated in 2023) was not used in this project since it was not yet published during data collection. The adaptation of the FNI framework to Canada’s food regulations is an ongoing work, and some aspects like the farming practices criteria were not entirely transposed. Another limit of using the FNI is the certain degree of subjectivity when evaluating ingredients that could be considered unexpected or unnecessary. Indeed, while a discussion was held between experts in food science and nutrition to classify ingredients under the different criteria, reaching a consensus view was not always a simple matter. While some ingredients such as palm or safflower oils are always considered unexpected or unnecessary according to the FNI, others such as added plant-based ingredients in sausages could be argued one way or another. That being said, the double coding procedure and the distinct criteria of the FNI do help in mitigating this limitation. Finally, the FNI only takes into consideration the source of the ingredients and not the quantity when calculating scores for each criterion. As such, a product with more sugar will have the same FNI score as the same one with less sugar, assuming the sugar content comes from the same source.

## Conclusion

In conclusion, findings from this innovative study suggest that the association between nutritional quality and degree of naturalness measured with the FNI in food products may vary according to the studied food category and the NP model used. Both concepts offer complementary information on a food product, therefore highlighting the importance of using the appropriate tools for ensuring proper evaluation of quality in food products.
